# The role of mouse strain differences in the susceptibility to fibrosis: a systematic review

**DOI:** 10.1186/1755-1536-6-18

**Published:** 2013-09-25

**Authors:** Louise Walkin, Sarah E Herrick, Angela Summers, Paul E Brenchley, Catherine M Hoff, Ron Korstanje, Peter J Margetts

**Affiliations:** 1 School of Medicine, Faculty of Medical and Human Sciences, University of Manchester, Oxford Road, Manchester M13 9PT, UK; 2 Manchester Institute of Nephrology and Transplantation, Manchester Royal Infirmary, Grafton St, Manchester M13 9WL, UK; 3 Baxter Healthcare, Renal Division Scientific Affairs, Baxter Healthcare Corporation, McGaw Park, Chicago, Illinois, 60015-4625, USA; 4 The Jackson Laboratory, 600 Main St, Bar Harbor, Maine 04609, USA; 5 Department of Pathology and Molecular Medicine and Division of Nephrology, McMaster University, 1200 Main St West, Hamilton, Ontario, L8S4L8, Canada; 6 3.107 Blond McIndoe Laboratory, Stopford Building, Oxford Road, Manchester M13 9PT, UK

**Keywords:** Mouse strain, Genetics, Fibrosis, Wound healing, Genetic susceptibility

## Abstract

In humans, a number of genetic factors have been linked to the development of fibrosis in a variety of different organs. Seeking a wider understanding of this observation in man is ethically important. There is mounting evidence suggesting that inbred mouse strains with different genetic backgrounds demonstrate variable susceptibility to a fibrotic injury. We performed a systematic review of the literature describing strain and organ specific response to injury in order to determine whether genetic susceptibility plays a role in fibrogenesis. Data were collected from studies that were deemed eligible for analysis based on set inclusion criteria, and findings were assessed in relation to strain of mouse, type of injury and organ of investigation. A total of 44 studies were included covering 21 mouse strains and focusing on fibrosis in the lung, liver, kidney, intestine and heart. There is evidence that mouse strain differences influence susceptibility to fibrosis and this appears to be organ specific. For instance, C57BL/6J mice are resistant to hepatic, renal and cardiac fibrosis but susceptible to pulmonary and intestinal fibrosis. However, BALB/c mice are resistant to pulmonary fibrosis but susceptible to hepatic fibrosis. Few studies have assessed the effect of the same injury stimulus in different organ systems using the same strains of mouse. Such mouse strain studies may prove useful in elucidating the genetic as well as epigenetic factors in humans that could help determine why some people are more susceptible to the development of certain organ specific fibrosis than others.

## Review

Genetic susceptibility is thought to be an important risk factor for many diseases [1]. Many conditions do not appear to be induced entirely by environmental factors and so genetic profiles have been studied to determine why some individuals are more susceptible to particular diseases. Whole-genome studies have provided a useful insight into how gene patterns may influence the development and/or progression of certain pathological pathways, including fibrosis [2]. This pathological end stage of tissue repair is characterised by excess extracellular matrix (ECM) deposition due to increased production and/or an imbalance in turnover often resulting in functional damage in tissues and organs [3,4]. Animal models have shown that the fibrotic response to various injurious agents differs as a result of genetic background [5,6]. In particular, different strains of rodents vary in their susceptibility to scarring and fibrotic pathologies and evidence suggests that such strain susceptibility may be organ specific [7,8].

The majority of genomic studies used to identify susceptibility pathways involved in disease pathologies have been performed in the mouse. Mice provide a suitable model for the study of human genetics because more than 95% of the genome is identical. Mice are also easy to breed with a short generation time and a short life span allowing direct study of disease development and can accurately eludicidate how genetic sequences may vary in different strains and result in susceptibility to fibrotic pathologies. Quantitative trait locus (QTL) analysis is a common genetic approach that has been used in the past two decades to identify loci involved in differences in fibrotic response between inbred strains of mice [9]. In this approach, two inbred strains that differ in fibrotic response are crossed and the F1 offspring are either mated together (intercross) or to one of the parental strains (backcross). The resulting second generation is phenotyped and genotyped for DNA markers covering the genome so that linkage analysis can be performed and loci identified. To date, the Mouse Genome Database through The Jackson Laboratory, lists 24 QTL in mice associated with fibrosis of which all but 3 are phenotypes in the lung. These QTL span large genetic regions often containing hundreds of genes and identifying the causal gene has been a major challenge. However, new genetic approaches like genome-wide association mapping [10] and the use of Collaborative Cross [11] and Diversity Outbred [12] mice all provide higher mapping resolution that should improve identification. Quantitative trait loci analysis can be time consuming and can only analyse two different mouse strains at a time. Alternatively, haplotype association mapping (HAM) is a phenotype-driven approach that requires genotyping to be performed only once and is able to achieve a higher resolution compared with other approaches [13].

The mouse genome is easy to manipulate to allow diseases to be modelled in transgenic animals when the ‘causative’ genes have been determined [14]. In the current study, we summarise the evidence from murine studies performed to assess fibrosis in different organs, and we discuss the influence of mouse strain on the susceptibility to fibrosis in these organs.

### Overview of fibrosis

Tissue repair is a normal response to injury and involves an orchestrated series of events with the end stage being extracellular matrix (ECM) remodelling leading to scarring/fibrosis [15]. Initial haemostasis and inflammation events are followed by granulation tissue formation with the influx and proliferation of fibroblasts and angiogenesis, restoration of epithelial integrity and subsequent ECM deposition and remodelling with apoptosis of myofibroblasts as part of the resolution phase [16,17]. Fibrosis usually develops in response to repeated injury and chronic inflammation and is characterised by excess deposition and accumulation of ECM components often leading to a loss in tissue function. The development of a fibrotic phenotype is generally thought to follow the same pathways as wound healing but due to changes in the cellular and molecular mechanisms and defective resolution of repair events the response becomes extended and often irreversible [18]. Characteristic fibrotic features often include epithelial cell hyperplasia, accumulation of inflammatory cells, and persistence of myofibroblasts with the extensive deposition of excess ECM components. The presence of inflammatory cells and production of a multitude of growth factors and cytokines drives and exacerbates the synthesis of ECM. In combination, reduced degradation and turnover due to an imbalance of matrix metalloproteinases (MMPs) with tissue inhibitors of metalloproteinases (TIMPS), results in a net matrix accumulation. Although a number of fibrotic mediators have been identified, transforming growth factor-β (TGF-β) plays a central role in ECM deposition and subsequent remodelling events and remains a key therapeutic target [19].

The development of many types of organ fibrosis has been investigated using mouse models however there are clear differences in the severity of the response to injurious agents between various strains of mice (Table 1). It is also apparent that particular strains may be susceptible to fibrosis in one organ but resistant to fibrosis in other organs. It is therefore possible that susceptibility to fibrosis may not just be strain specific but also organ specific. We have performed a systematic review to determine the validity and inform on the appropriateness of using a particular mouse strain to model a human organ-specific fibrotic pathology. Such information may also be beneficial in determining which genetic signatures are associated with susceptibility to fibrosis and have translational relevance to identify individuals who may be susceptible to the development of a fibrotic phenotype in a particular organ following injury or disease.

**Table 1:** Summary of the various types of organ fibrosis and associated mouse strain susceptibility

**Strain**	**Susceptibility**	**Fibrosis type**	**Reference**
**C57BL/6J**	Susceptible	Pulmonary	[7]
		Hepatic	[20]
		Renal	[8]
	Resistant	Hepatic	[21]
**C57BL/6**	Susceptible	Pulmonary	[22]
		Intestinal	[23,24]
		Hepatic	[25]
		Renal	[26]
	Intermediate	Hepatic	[21]
	Resistant	Renal	[27]
		Cardiac	[28]
			[29]
**BALB/c**	Susceptible	Hepatic	[21]
	Resistant	Pulmonary	[22]
		Hepatic	[25]
		Renal	[26]
**BALB/cJ**	Susceptible	Hepatic	[20]
**C3Hf/Kam**	Resistant	Pulmonary	[30]
		Intestinal	[23,24]
**C3H/HeJ**	Intermediate	Hepatic	[20]
**A/J**	Susceptible	Cardiac	[29]
	Resistant	Hepatic	[20]
		Pulmonary	[31]
**DBA/2**	Intermediate	Pulmonary	[22]
**DBA/2J**	Intermediate	Hepatic	[20]
**MRL/MpJ**	Resistant	Renal	[32]
		Cardiac	[33]
	Susceptible	Cardiac	[34]
**MRL/MpJ**^**lpr/lpr**^	Susceptible	Renal	[32]
**129S1/SvJM**	Susceptible	Renal	[8]
**129Sv**	Resistant	Renal	[27]
**129S3**	Susceptible	Renal	[28]
**Swiss**	Intermediate	Pulmonary	[22]
**AKR/J**	Resistant	Hepatic	[20]
**CD1**	Susceptible	Renal	[27]
			[28]
**FVB/NJ**	Resistant	Hepatic	[20]
**Col4A3**^**-/-**^	Susceptible	Renal	[32]

### Systematic review of mouse strain differences in the susceptibility to fibrosis in different organs

Searches were performed online using MEDLINE from 1980 to July 2013 limited by ‘animals’ and routinely using two sets of keywords ‘fibrosis AND genetics’ and ‘fibrosis AND strain’ with a third set of keywords as follows: lung OR pulmonary; hepatic OR liver, renal OR kidney; intestinal OR bowel; and cardiac OR heart. Studies were considered eligible for analysis if they (1) compared at least two different strains of mice, (2) quantified a fibrotic indicator in a particular organ(s) as an outcome measure, and (3) used a particular type of injurious agent. From the 44 studies found to fit the criteria, a total of 22 were analysed for pulmonary fibrosis, 8 for hepatic fibrosis, 6 for renal fibrosis, 3 for intestinal fibrosis and 5 for cardiac fibrosis.

#### Mouse strain differences in susceptibility to pulmonary fibrosis

Pulmonary fibrosis (PF) is a complex disease with excessive deposition of collagen and other ECM components in the pulmonary interstitium preventing normal gaseous exchange [35,36]. The disease has an incidence of approximately 7 to 10 in 100,000 people with a high mortality 2 to 5 years after diagnosis [36,37]. Inflammatory, apoptotic and remodelling (MMPs/TIMPs) events have all been implicated in pathogenesis [38-40]. Idiopathic pulmonary fibrosis, the most common form, has an unknown aetiology. It is recognised that bleomycin, a chemotherapeutic agent used clinically in cancer patients, causes some patients to develop PF as a side effect [40,41]. Pulmonary fibrosis patients demonstrate a large amount of symptomatic variability, which has encouraged the exploration of possible genetic influences [42].

Intra-tracheal delivery of bleomycin in rodents causes lung damage through direct DNA strand breakage and generation of free radicals inducing oxidative stress [43]. Radiation is another known causative agent, and this has also been used in some animal models of pulmonary fibrosis. Using the search terms described, 22 studies fitted the criteria with bleomycin and radiation as injurious agents, 12 used bleomycin as the injurious agent and 10 used radiation as the injurious agent. Results suggest that different mouse strains were either susceptible or resistant to bleomycin-induced lung fibrosis. However, the dose of bleomycin, number of applications, as well as the route of administration, was not standardised, which may impact on the extent of the fibrotic response in different studies.

In one study, a single intra-tracheal dose of bleomycin was used and the fibrotic response determined by assessing collagen synthesis and deposition. C57BL/6 mice were most susceptible, DBA/2 and Swiss mice were intermediate whereas BALB/c were most resistant to lung fibrosis [22]. In another study, mRNA expression of a panel of cytokines linked to fibrosis was assessed after a single dose of bleomycin in two mouse strains, C57BL/6J and BALB/cBy [44]. TGF-β1 gene expression was found to be threefold lower in resistant BALB/cBy mice in comparison to a sevenfold increase in susceptible C57BL/6J mice. In addition, BALB/cBy mice were found to have fourfold higher levels of IL-1β mRNA than C57BL/6J mice suggesting a possible protective effect of IL-1β. However, no strain difference was found with TGF-β2, IFN-γ, IL-2, IL-3 or IL-4 mRNA levels but gene expression for IL-4 receptor was higher in C57BL/6J suggesting this receptor may play a role in the susceptibility to pulmonary fibrosis.

Strain differences between C57BL/6 and BALB/c mice were also confirmed in another study following a single intra-tracheal dose of bleomycin as collagen deposition was higher in C57BL/6 mouse lungs compared with BALB/c lungs at 16 days and had increased further at 30 days after administration. Connective tissue growth factor (CTGF), a downstream mediator of TGF-β, was also found to be upregulated in C57BL/6 mice but not in the BALB/c strain [45]. The strain difference may be due to a reduced production of bleomycin hydrolase activity resulting in limited oxygen free radical induction in the lungs of BALB/c mice [46]. Another possibility to explain the strain differences in response to bleomycin may be less DNA breakage in BALB/c lungs [47].

In a separate study, after a single intra-tracheal injection of the bleomycin, C57BL/6 mice showed increased expression for MMP-2, MMP-7, MMP-9 and MMP-13 at both 7 and 14 days after administration compared with the BALB/c strain. TIMP-1, an endogenous inhibitor of MMPs, was upregulated in the lungs of C57BL/6 mice but not in BALB/c mice. Experimental reduction of MMP expression did not, however, alleviate fibrosis in C57BL/6 mice and there was no change in TIMP-1 levels, which led to the conclusion that although expression varies between the strains, TIMP-1 overexpression does not obviously alter the development of lung disease in mice [48].

Another group also identified BALB/c mice as being fibrosis resistant and C57BL/6 mice as being fibrosis susceptible following adenovirus delivery of active TGF-β1 [39]. In a subsequent study, the same group showed that adenoviral lung delivery of the downstream mediator of TGF-β1, connective tissue growth factor (CTGF) in combination with a single intra-tracheal injection of bleomycin caused BALB/c mice to develop a fibrotic phenotype similar to that of the C57BL/6 strain with a persistent upregulation of TIMP-1 [49].

Although the exact mechanism underlying the difference in fibrotic response between these two strains of mice is unclear, a number of other theories have been proposed, including a reduced production of bleomycin hydrolase activity as mentioned earlier [46]. Furthermore, there is reduced poly(ADP-ribose) polymerase activity and, hence, less DNA breakage in BALB/c lungs compared with C57BL/6 mice after bleomycin exposure [47,50].

A further study investigated the effect of mouse strain variation on the expression of genes associated with apoptosis in bleomycin-induced pulmonary fibrosis. A single dose of bleomycin was administered to the trachea of two strains of mice, NMRI and C57BL/6, and expression of BAX, an apoptotic gene that down-regulates BCL-2, a death repressor gene, was analysed in lung tissue as was collagen content. After 14 days, although the fibrotic response appeared similar in both strains, BCL-2 was upregulated in myofibroblasts of NMRI mouse lungs but not in C57BL/6. BAX was upregulated in alveolar epithelial cells of both strains but was only downregulated in myofibroblasts of C57BL/6 lungs, suggesting that the regulation of apoptotic genes may be specific to cell type in the different strains of mice [51].

A later study used both a single dose and a repetitive exposure to bleomycin with a three-time weekly injection by intra-tracheal administration to encourage a chronic response in C57BL/6J mice and DBA/2 mice [52]. Collagen content of the lung at 3 and 6 weeks after the last injection was measured as a fibrotic readout. Interestingly, bleomycin administration induced a Th2 response in both the Th-1 biased C57BL/6J mice and Th-2 biased DBA/2 mice. Fibrotic changes were evident in both strains at early time points but were prolonged only after repeated exposures in DBA/2 mice. However, when the repair process was experimentally delayed in C57BL/6J mice, these mice also showed prolonged fibrosis after multiple bleomycin exposures, leading to the conclusion that genetic background and type of injury are both relevant in the persistence of lung fibrosis [52].

Haston and colleagues used a subcutaneous osmotic mini-pump to deliver bleomycin to the lungs of C57BL/6J and C3Hf/Kam mice as an alternative to repeated injection [7]. The fibrotic phenotype was measured by assessing collagen deposition in the lungs by histological assessment up to 8 weeks after treatment. Examining the offspring in the F1 and F2 generations after inter-crossing between strains showed that mice with a strong C57BL/6J background developed substantial fibrosis, which was more obvious in male mice whilst mice with the C3Hf/Kam background did not show a response [7].

In a follow-up study, the same group went on to map loci linked to the phenotypic difference in the response to bleomycin between the two mouse strains and found two regions named: *Blmpf1*, the MHC complex on chromosome 17, and *Blmpf2* on chromosome 11 in males only [30]. Using linkage analysis, the group showed that inheritance of B6 alleles at either locus increased the fibrosis phenotype of B6 x C3H cross F2 mice. In a follow-up study, loci regulating bleomycin-induced lung fibrosis were mapped in fibrosis-prone C57BL/6J mice compared with the fibrosis-resistant A/J strain [31]. Genetic analysis found 3,304 genes that were differentially expressed between the two strains, with 246 linked to susceptibility to lung fibrosis and many of these linked to heparin binding and ECM deposition pathways [31]. Additional gene expression and sequencing studies using a fibrosis-prone C57BL/6J and fibrosis-resistant C3Hf/KAM mouse cross examined susceptibility genes linked with bleomycin-induced pulmonary fibrosis [53]. Results showed that 1,862 genes or expressed sequence tags (ESTs) were differentially expressed between treated C57BL/6J and C3Hf/KAM mice, and 67 of these mapped to *Blmpf1* or 2. This group of genetic elements includes genes relating to apoptosis, immune regulation and oxidative stress. Assessing the correlation among other inbred strains showed that 36% of the sequence variations between these strains can predict for the well-known bleomycin-induced pulmonary fibrosis susceptibility of the DBA mice and the resistance of the A/J mice [53]. *In vitro* studies have reported the same pattern of strain specific response in cultured cells. For instance, the response of specific mouse strain-derived lung fibroblasts to asbestos rather than bleomycin as the injurious agent showed that lung fibroblasts from the 129 mouse strain proliferated less than those from the C57BL/6 and SJL strain and showed a reduced response in terms of cell proliferation to PDGF [54].

Radiation therapy in the thoracic cavity produces a similar fibrotic lung phenotype to bleomycin treatment. Damage to lung tissue by radiation may produce an inflammatory pneumonitis reaction or chronic fibrosis with ECM accumulation. Several groups have shown differences in the development of pulmonary fibrosis following radiation damage using different mouse strains.

In response to a single dose of radiation to the thorax, C57BL/6 mice were shown to be more prone to pulmonary fibrosis and C3H/HeJ and CBA/J mice more resistant after 14 to 25 weeks [55]. Another study compared C57BL/6 mice and C3H/HeJ mice exposed to two doses of radiation to the thorax and measured gene expression of collagen I, III and IV, fibronectin, and TGF-β1 and TGF-β3 at 8, 16 and 26 weeks. Expression was shown to be unaltered in the resistant C3H/HeJ strain until 26 weeks after radiation, whereas with the C57BL/6 mice, increased levels of collagen I and fibronectin were seen in the lungs at all time points [56]. In a further study by the same group, IL-1 alpha and TNF-alpha were proposed to play a role in susceptibility to radiation induced pulmonary fibrosis however IL-1 beta may have a protective role [57].

Iwakawa and colleagues investigated the effect of two doses of radiation on two strains of mice (C57BL/6J and C3H/HeMs) and histological analysis demonstrated that C3H/HeMs mice are able to clear ECM more rapidly after radiation-induced inflammation compared to C57BL/6J [58]. Gene-expression analysis suggested that *Ltf*, *Ifi202a*, *Ill8*, *Per3*, *Mmp12*, *Cap1* and *Rad51ap1* are linked with mouse strain differences in the fibrotic response to radiation. Ao and colleagues also analysed the difference in cytokine expression after radiation damage in fibrosis-sensitive (C57BL/6) and resistant (C3H) mice. An increase in cytokine levels occurred earlier in the C57BL/6 mice and was also more enhanced supporting the fibrotic phenotype [59]. A further study assessed the lung fibrotic responses of C57BL/6 mice to three doses of radiation comparing C57/L and CBA mouse strains. The most significant finding was the difference between the two different C57 strains, as C57/L mice were more sensitive and developed early pneumonitis and fibrosis at 3 to 4 months while C57BL/6 mice presented with a delayed response at 6 to 9 months [60]. This finding was also confirmed in another study suggesting that the C57/L strain is a better fibrotic sensitive strain than the CBA strain to use in radiation studies [61]. Following whole-body radiation, C57BL/6J mice were more susceptible to fibrosis and displayed higher TGF-β1 mRNA on day 9 compared to C3H/J. There was no TGF-β1 gene expression production in controls or in both groups by day 56 [62].

Haston’s group administered two doses of radiation in two strains of mice, sensitive C57BL/6J and resistant C3Hf/Kam, and in their intercross F1 and F2 offspring, and data suggested that approximately 38% of the phenotypic differences were linked to a few genetic factors [63]. Using phenotypic data, a genome-wide linkage scan found evidence for a quantitative trait locus (QTL) on chromosome 17, which appeared to influence susceptibility to radiation-induced pulmonary fibrosis similar to that found with bleomycin induced fibrosis. This suggests that that area of chromosome 17 may contain a lung injury gene [30].

The identification of fibrosis genes in humans has been extensively studied, and a number of polymorphisms relating to susceptibility to idiopathic pulmonary fibrosis have been found. However, despite the developments in mouse strain studies, there have not yet been any QTLs found in the humans to help determine those at risk of developing lung fibrosis. More study into the mouse genome and potential candidate genes will eventually allow study in the human condition into the same candidate genes to establish susceptibility to lung fibrosis.

#### Mouse strain differences in susceptibility to hepatic fibrosis

Chronic liver disease, such as cirrhosis and hepatitis, results in the development of liver fibrosis and is a leading cause of death [64]. All chronic injuries to the liver, whether viral, immune, metabolic, obstructive or toxic, lead to fibrosis regardless of the injurious agent or the route of injury [65]. When injury occurs, a heterogenous population of hepatic myofibroblasts are primarily responsible for the fibrotic response [66]. Myofibroblasts come from a number of possible sources including activation of mesenchymal cells such as fibroblasts and hepatic stellate cells, via EMT from epithelial cells or by recruitment from the bone narrow. With an increase in ECM deposition, normal liver tissue becomes replaced by scar tissue with changes in the organisational structure that can lead to organ failure.

The most common experimental model uses mice to study hepatic fibrosis induced by diet changes or administrations of carbon tetrachloride (CCl_4_), which is a hepatotoxin and causes hepatocyte death and liver degeneration [67]. Using the search terms described, eight studies fitted the criteria with carbon tetrachloride used in two studies and a specific diet used to induce fibrosis in six studies.

From these studies, there was substantial evidence to suggest that certain mouse strains were either susceptible or resistant to hepatic fibrosis. In particular, BALB/c mice were fibrosis-susceptible which is in contrast to findings for lung fibrosis [21]. BALB/c mice were found to be susceptible to fibrosis induced by CCl_4_ whereas C57BL/6J mice were fibrosis-resistant. Interestingly, even though BALB/c mice were found to be susceptible to fibrosis induced by CCl_4_ they were resistant to fibrosis induced by a carcinogenic diet. Such findings suggest that the route and type of injury and the subsequent pathways activated are linked to susceptibility. As with mouse lung fibrosis studies, the dose of injurious agent (CCl_4_), the number of administrations, and the route of administration was found to vary in the different studies making a determination of impact of the various agents on the extent of the liver fibrosis difficult.

Shi and colleagues induced liver injury in two mouse strains by four gavage administrations of CCl_4_ at 7-day intervals for 28 days and tissue was collected 7 days after the final dose was administered [21]. In contrast to lung fibrosis findings, BALB/c mice showed severe liver fibrosis at day 7 whereas the C57BL/6 mice developed minimal fibrosis with reduced ECM expression and lower levels of pro-collagen I mRNA detected [21]. Furthermore, BALB/c mice showed a Th2 response whereas C57BL/6 mice showed a Th1 response and the authors propose a role for immune modulation in liver fibrosis and that the T cell cytokine profile may regulate the fibrotic response to injury.

A wider examination of strain differences used a protocol involving two intraperitoneal (IP) CCl_4_ injections per week, for either 6 or 10 weeks and the fibrotic response was assessed 2 days after the last dose in seven strains of mice: A/J, AKR/J, BALB/cJ, C57BL/6J, C3H/HeJ, DBA/2J and FVB/NJ [20]. BALB/cJ mice were again the most susceptible strain with significantly higher levels of liver hydroxyproline as a measure of collagen compared to AKR/J, A/J and FVB/N mice. C57BL/6J mice developed an intermediate response; however, Shi *et al*. [21] showed that C57BL/6J mice are resistant to CCl_4_ induced fibrosis, suggesting that the route of injury or administration regime may affect the fibrotic response Further QTL analysis of intercross F1 progeny found that liver fibrosis was linked to a susceptibility locus on chromosome 15 known as hepatic fibrogenic gene 1 (*hfib1*) [20].

A carcinogenic diet was used in a second model of liver injury to compare the fibrotic response between BALB/c and C57BL/6 mice [25]. The choline-deficient, ethionine-supplemented diet induced oval cell proliferation to mimic liver injury. Oval cells are essentially the ‘stem cells’ of the liver that can transform into any of the many cell types found in the liver [68]. Mice were fed a choline-deficient chow and ethionine-supplemented water, and the fibrotic response in the liver was measured over 21 days. In this study, BALB/c mice showed a reduced response compared with C57BL/6 mice suggesting that the livers of mice with a genetic background similar to C57BL/6 may be more susceptible to a fibrotic response [25]. The contradicting results between the two models could be explained by the fact that the two models induce different types of liver injury and BALB/c mice may be susceptible to one injurious pathway but protected from another. There may be certain pathways that are activated in response to a specific injury, causing particular strains to be susceptible to one type of injury but less susceptible to another.

Millward and colleagues had shown previously that there were QTLs in the chromosome substitution strain (CSS)-17 mouse strain that made it resistant to obesity and liver steatosis whilst on a high fat diet [69]. These were QTL 13 (*Orbq13*) and QTL 15 (*Orbq15*) A later study by the group compared the CCS-17 strain to C57BL/6 and A/J mice and used a diet induced or CCl_4_ injury. In comparing these strains they determined that *Orbq13* led to resistance to liver injury and *Orbq15* led to susceptibility to liver injury. C57BL/6 and A/J strains sequencing data showed that the *Orbq* genes contained small nucleotide polymorphisms within the gene. They were able to identify candidate genes that modulate genetic susceptibility to alcoholic steatohepatitis [70].

Another study assessed the effect of a methionine-choline deficient diet on seven inbred strains; A/J, AKR/J, BALB/c, C57BL/6J, DBA/2J, C3H/HeJ and 129x1/SvJWT. A/J mice demonstrated the highest steatohepatitis and gene loci were identified on four chromosomes, particularly chromosomes 2 and 15 in regions that had previously been identified for liver fibrosis [71]. Other studies have assessed the susceptibility between two strains - C57BL/6 N and C3H/HeN - using the same model of a methionine-choline deficient diet. C57BL/6 N livers were shown to have the highest inflammation, steatosis and lipid contents and a higher degree of fibrosis, showing that this strain is more susceptible to diet-induced liver fibrosis [72]. Another study used a methyl-deficient diet and compared C57BL/6J and DBA/2J mice. DBA/2J demonstrated more severe changes and had a greater extent of fibrosis genes in their livers. There were also changes in miRNA and the protein levels of their targets [73].

Genome-wide association studies (GWAS) assess the association between genetic variants across the whole genome and a known disease of interest. Several have been performed in patients with hepatic disease with particular genes being determined as risk genes. In terms of hepatic fibrosis, seven genes have been identified as being risk genes with single nucleotide polymorphisms (SNPs) also being identified [74]. The combination of these investigations with inbred mouse genomic analysis studies may determine susceptibility to hepatic disease in humans.

#### Mouse strain differences in susceptibility to renal fibrosis

Diabetic nephropathy and chronic kidney disease (CKD) are two leading causes of end stage renal disease (ESRD). They both involve the progressive loss of kidney function leading to renal failure and eventual death. CKD is mainly caused by diabetes and hypertension and characterised by reduced glomerular filtration rate (GFR) proteinuria, interstitial fibrosis and anaemia [26]. Diabetic nephropathy has similar features with reduced renal function and an increased risk of cardiovascular disease. Histological features include glomerular hypertrophy, thickening of the glomerular basement membrane, matrix expansion and interstitial fibrosis. Renal fibrosis is implicated in both of these conditions and is characterised by glomerular injury, tubular atrophy and tubule-interstitial fibrosis, which manifests clinically as proteinuria, which can progress to end stage renal disease (ESRD) [26].

A well-described mouse model of renal fibrosis, involves unilateral ureteral obstruction (UUO), which mimics the effects of diabetes and CKD [75]. Other models include using IP injection of bovine serum albumin (BSA), which increases the amount of protein in the urine and induces fibrosis. Another method involves inducing diabetes to cause renal injury and eventual fibrosis. Using the search terms described, six studies fitted the criteria with unilateral ureteral obstruction used in two studies, IP injection of BSA used in one study, induction of diabetes used in one study, IP injection of recombinant human bone morphogenetic protein (rhBMP) used in one study and induction of hypertension used in one study. From these studies, there was substantial evidence to suggest that certain mouse strains were either susceptible or resistant to renal fibrosis. However, the only strain that was examined in two different models was the C57BL/6 mouse, which was found to be resistant to fibrosis in both the diabetes induced and BSA administration studies [27]. However, as with pulmonary and hepatic fibrosis studies, the route of administration was found to vary greatly between models and was likely to impact the extent of fibrosis. It is possible that susceptibility to fibrosis is linked to the type of injury induced and may affect the patterns seen between the strains. The fibrotic response in the kidney to IP injections of bovine serum albumin (BSA) at 10 mg per gram of body weight for 6 weeks was measured in two strains of mice. 129S1/svImJ mice were found to be susceptible to renal fibrosis and C57BL/6 mice were found to be resistant [8].

However, a previous study reported that both these strains were resistant to fibrosis induced by diabetic nephropathy, with outbred CD1 mice being more susceptible to renal fibrosis [27]. Diabetes was induced by a single IP injection of streptozotocin at 200 mg/kg and mice were sacrificed at 3.5 months and 6 months. It is possible that the time frame in which the fibrotic response was measured, as well as the route of injury, may have affected the outcome. It also suggests that there may be a grading in the response between strains.

Susceptibility to chronic kidney disease (CKD) also demonstrates mouse strain differences in fibrotic response. Chronic renal injury can be induced in mice by clamping the renal arteries, UUO or folic acid administration [28]. Interstitial fibrosis and glomerulosclerosis developed in both CD1 and 129S3 mice after 12 weeks. However, C57BL/6 mice, at 16 weeks, did not develop these symptoms. Pharmacological manipulation of blood pressure with angiotensin II affected susceptibility as C567BL/6 mice did develop fibrosis once their blood pressure had been increased. Hypertension is a factor that has not yet been investigated in other organs and may be a contributing factor in the development of organ fibrosis.

C57BL/6 and BALB/c mice were used to study susceptibility to CKD by inducing fibrosis through ureteral obstruction by clamping of the ureters. C57BL/6 mice showed pronounced inflammation and a development towards fibrosis, whereas BALB/c mice showed a reduced response, with damaged renal tissue returning to normal after injury [26]. This is in contradiction to the patterns shown in the liver where BALB/c mice were susceptible to fibrosis, but follows similar results in the lung. Zeisberg and colleagues induced renal injury by administration of rhBMP-7 via IP injection, MRL/MpJ, and MRL/MpJ^lpr/lpr^ mice were used to assess strain differences [32]. MRL/MpJ^lpr/lpr^ mice were shown to be susceptible whereas the MRL/MpJ mice were shown to be resistant to renal fibrosis post-injection. MRL/MpJ mice have a regenerative capacity to heal ear-hole punches completely and rapidly without scarring [76]. It would be interesting to determine how this strain would respond in response to injuries that have been described in other organs to determine whether regenerative capacity is linked to the fibrotic response.

Another study assessed the susceptibility of four mouse strains: C57BL/6 and 129/Sv and F1 and F2 crosses of C57BL/6 and 129/Sv. Mice were uninephrectomised and hypertension was induced. 129/Sv mice demonstrated increased blood pressure, glomerulosclerosis and interstitial fibrosis in comparison to C57BL/6 mice. F1 and F2 crosses showed an intermediate response [77]. There have been limited studies to assess strain susceptibility to kidney fibrosis. More studies are necessary to develop the understanding of which strains are susceptible and resistant to find potential QTLs and candidate genes that can be related back to the human condition of kidney fibrosis.

#### Mouse strain differences in susceptibility to intestinal fibrosis

Intestinal fibrosis is a complication associated with inflammatory bowel disease (IBD) and is associated with both Crohn’s disease and ulcerative colitis. Crohn’s disease can affect the entire thickness of the intestinal wall and normally requires surgery as a management therapy. Acute injury of the intestinal mucosa is followed by a process of normal wound healing to restore tissue function. Deposition of excess ECM causes thickening of the mucosa and narrowing of the lumen, leading to obstruction of the bowel [78].

In mouse models, intestinal fibrosis has been induced by either a single dose of radiation to the colorectum with set or varying lengths of tissue exposed or by worm parasite-induced or intra- rectal administration of trinitrobenzene sulfonic acid [79]. Then, the fibrotic response is measured after 6 weeks. Using the search terms described, three studies fitted the criteria with irradiation used in two studies and treatment with the worm *Trichuris muris* (*T.muris*) used in one study. There was evidence to suggest that mouse strains were either susceptible or resistant to intestinal fibrosis. In particular, C57BL/6 mice were fibrosis-susceptible whereas C3Hf/Kam mice were resistant to irradiation-induced fibrosis [23]. However as with the pulmonary fibrosis studies, the dose of radiation was the same but the lengths of tissue that were exposed to radiation varied, and this is likely to affect the extent of fibrosis.

Where the colon was irradiated once, C57Bl/6 mice showed greater bowel obstruction from day 60 in comparison to C3Hf/Kam mice. There was a threshold in the length of colorectum that was irradiated before obstruction was demonstrated, a threshold that was greater in the C3H mice [23]. The same group later repeated this study but examined obstruction between 6 hours and 120 days as opposed to the previous study [24]. C57BL/6 mice showed increased evidence of hyperplastic crypts and fibrosis in the intestinal mucosa in comparison to the C3H mice. Fibrosis was seen in the C57BL/6 strain at 75 days, which was much earlier than in the C3H mice. Significant strain-dependent differences were seen in the architecture of the colon between the strains but not in the levels of TGF-β1, 2, 3 or TNF-α [24]. These results support the findings demonstrated in models of pulmonary fibrosis due to irradiation. It is also apparent that particular doses may be harmless but once a dosage threshold has been reached, this may be detrimental to particular strains.

A recent study assessed two strains of mice, a susceptible AKR/OlaHsd and a resistant BALB/cOlaHsd and their F1 offspring after treatment with *T.muris* for 35 days to assess potential QTLs. They identified seven potential QTL and four candidate genes for susceptibility to colitis, *FCgR1*, *Ptpn22*, *RoRc* and *Vav3*[80]. However, these data have not yet been mapped to the human condition of intestinal fibrosis.

#### Mouse strain differences in susceptibility to cardiac fibrosis

Cardiac fibrosis is a known causative factor of cardiac failure. Injury to the heart may be caused by insults that result in a significant loss of cardiomyocytes such as myocardial infarction or do not lead to extensive cardiomyocyte loss, such as pressure overload, hypertrophic cardiomyopathy, dilated cardiomyopathy, repetitive ischaemic episodes, and diabetes or obesity-induced cardiomyopathy [81]. Resulting fibrosis with excessive accumulation of ECM proteins in the heart causes increased mechanical stiffness and disruption to electrical impulses that control the contraction of the heart and leads to an increased chance of arrhythmias. After myocardial infarction, normal cardiac tissue heals by forming a scar rather than regenerating new tissue. Cardiac fibroblasts play a key role in the scarring process, and, due to their different sources in the heart, they are a highly heterogenic cell population. It is proposed that cardiac fibroblasts can derive either from resident fibroblasts; from endothelial cells via an endothelial-mesenchymal transition; or from bone marrow-derived circulating progenitor cells, monocytes and fibrocytes [82,83].

Cardiac fibrosis in animal models can be induced by a physical injury such as cryogenic damage or by coronary artery ligation to produce myocardial infarction. Using the search terms described, five studies fitted the criteria with two inducing infarct and three using a physical injury to induce fibrosis. As with the other types of organs, certain mouse strains were either susceptible or resistant to cardiac fibrosis but as with the other fibrosis studies, the route of injury was found to vary and is likely to have had an impact on the extent of the fibrosis.

In one particular study, five strains of mice were subjected to coronary artery ligation and myocardial infarction assessed up to 28 days post-injury [84]. Infarct rupture occurred between 3 and 6 days and was most frequent in 129S6 mice (62%), then by C57BL/6 (36%), FVB (29%), Swiss (23%) and lastly BALB/c (5%). Furthermore, 129S6 and C57BL/6 mice were shown to be fibrosis-susceptible whereas BALB/c mice were fibrosis-resistant but showed significant secondary thinning of the infarct area [84]. A strain of mouse, MRL/MpJ, which was also used in renal fibrosis studies, was found to have the capacity to regenerate cardiac tissue with normal function 60 days after a single myocardial infarction caused by cryogenic damage to the right ventricle. Scarring and cardiac fibrosis was markedly reduced in comparison with C57BL/6 mice [33]. However, contradictory findings were later produced by several other groups using both cryoinjury [34] and coronary artery ligation procedures [85].

These studies showed that both MRL/MpJ and C57BL/6 mice repaired damaged tissue with pronounced scarring after infarction with no significant difference seen between the two strains. The reason for this difference in findings remains unexplained given that there was no major discrepancy in the experimental protocols used. Faulx and colleagues used a different model of cardiac fibrosis involving administering six daily injections of the catecholamine, isoproterenol, which causes left ventricular hypertrophy independent of blood pressure [29]. Excessive deposition of ECM proteins was linked to the development of hypertension and myocardial infarction. A/J mice showed a greater hypertrophic response in comparison to C57BL/6J mice, which displayed greater levels of myocardial metabolic enzymes that may protect against the development of fibrosis [29].

Similarly to the other types of organ fibrosis, few studies have assessed strain susceptibility to cardiac fibrosis. Once again, more studies are necessary to identify potential candidate genes to develop a better understanding of the human clinical condition and to identify individuals that are susceptible to cardiac fibrosis.

## Discussion

This systematic review has shown that there are differences in susceptibility to develop fibrosis in various inbred mouse strains. These differences are also organ specific. It is possible that different types of injury used to induce fibrosis in each organ may be linked to different signaling pathways, which themselves may be linked to susceptibility. However, only a limited number of studies have included the same strains of mice or same type of injury, which would allow a thorough comparison of the development of fibrosis in different organ systems. From the studies analysed, there are many strains that were assessed only in relation to one type of organ pathology, with C57BL/6 being the most commonly used. It is therefore difficult to directly compare the organ responses or route of injury in multiple strains in relation to susceptibility to the development of fibrosis. The possible mechanisms involved in susceptibility and protection from fibrosis are complex as there are many highly interactive pathways involved. In part, some of the findings may relate to the ability to process and clear the injurious agent. For instance, high levels of bleomycin hydrolase have been found in mice that are ‘non-fibrotic’ after bleomycin-induced damage in the lung [7].

A study that was performed in humans showed that variation in the bleomycin hydrolase gene is associated with reduced survival after treatment with chemotherapy in patients with testicular germ cell cancer [86]. Future studies that assess potential QTLs while using different strains of mice may help to determine which patients undergoing chemotherapy may be susceptible to lung fibrosis. It is possible that a different profile of key factors, both at baseline and involved in the repair process, may be mouse strain-determined and dictate susceptibility or resistance to fibrosis. In hepatic fibrosis, the level of IL-4, which is responsible for promoting collagen synthesis, were higher in the susceptible mice than in the mice that were resistant [21]. Also, in the liver, production of hyaluronan was associated with the deposition of ECM, and levels of hyaluronan synthase 2, which catalyses the synthesis of hyaluronan, could be another potential factor determining mouse strain differences [20]. Our group investigated mouse strain differences in relation to susceptibility to peritoneal fibrosis using an intraperitoneal injection of adenovirus over expressing TGF-β1 [87]. The fibrotic response was graded between mouse strains with C57BL/6J mice showing a significant fibrogenic response and SJL/J mice being resistant. Both the DBA/2 and C3H/HeJ mice demonstrated an intermediate response. A possible reason for the strain difference in response may be linked to mesothelial layer integrity and, hence, susceptibility to EMT leading to fibrosis and delayed epithelial repair in C57BL/6J. Currently there have been no other investigations into the mechanisms of the strain difference in susceptibility to peritoneal fibrosis. Further assessments are necessary to determine the mechanisms and candidate genes responsible.

Mouse models provide a controlled environment to study the effects of alleles that have been identified as risk alleles in the clinic. Phenotypes measured in varying strains can act as a base for genetic studies to determine the variation that causes a specific phenotype to develop and predisposes an individual to a disease, in this case fibrosis. These mouse studies are complimentary to human association studies and allow the analysis firstly of the clinical disease but also of the genetic variants that are associated with the disease to be assessed in a controlled environment [88].

Organs in the same inbred strain of mouse share the same DNA sequence, yet exhibit widely differing structure and function, demonstrating major epigenetic control of gene expression in normal development and physiology. The observation that there are different susceptibilities of various organs to fibrosis within the same mouse strain also highlights epigenetic control as an important modulator of fibrotic response. Evidence that differential DNA methylation of genes in an organ within a strain (the organ methylome) controls susceptibility or resistance of that organ to fibrotic injury, is a recent and new hypothesis, which requires further examination. Already, experimental data has been presented that the fibrotic response is in part under epigenetic control in the kidney [89].

Tissue regeneration, like fibrosis, has also been linked to the genetic background of mouse strains (Table 2). In particular, one strain of mouse was able to fully regenerate injured tissue without scarring. One study compared two strains of mice - MRL/MpJ-Fas (MRL-F) and C57BL/6 - and found that MRL-F mice completely heal a hole punched into the ear within 4 weeks with complete restoration of tissue function in comparison to C57BL/6 mice, which healed only 30% of the lesion and produced scar tissue [90]. When studying the mice, three stages of the healing process were described: the initiation stage, the fast healing stage and the slow healing stage. At the fast healing stage, there was dramatic variation between strains, with an acceleration of up to four times in MRL versus C57BL/6 mice. Later studies showed a correlation between the breakdown of basement membrane proteins, inflammatory response and increased protease/anti-proteases with ear-hole closure [91]. Similar studies using MRL/MPJ, C57BL/6 and BALB/c mice found that all strains healed similarly in the first 3 days and all mice, except the MRL/MPJ, showed an increase in the wound area up to 2 weeks after initial wounding possibly due to increased tissue necrosis. Regeneration of amputated digit tips was also investigated in MRL mice, and digits were found to re-grow with partial reformation of nails significantly more than the other strains, C57BL/6 and DBA/2 mice [92].

**Table 2:** Regeneration and strain susceptibility

**Wound type**	**Strain**	**Resistance**	**Reference**
**Skin**	MRL/MPJ	Fast Healer	[93]
**Ear hole punch**	C57BL/6	Intermediate Healer	[91]
	BALB/c	Slow Healer	
	MRL	Fast Healer	
	C57BL/6	Slow Healer	
**Digit tip Amputation**	MRL	More regrowth	[5,92]
	C57BL/6	Reduced regrowth	
	DBA/2	Reduced regrowth	

## Conclusion

This review discusses the increasing evidence that genetic susceptibility plays a role in regulating the development of fibrosis and also in regeneration in various tissues and organs. Various strains of mice respond differently to fibrosis induced by different agents but interestingly in varying organs. The possibility that there may be organ specific responses that vary between different strains of mice highlights the complex interaction between the genome and epigenome. More studies need to be performed with standard experimental procedures using the same inbred mouse strains to identify true genetic control of susceptibility and resistance to the stimulus. Then, within a strain, relating organ susceptibility or resistance to the epigenome of that organ (methylome), histone lysine code and miRNA signature may explain the mechanisms and pathways responsible for the fibrotic or non-fibrotic phenotypes that are observed in the various strains. Better understanding of the interaction between the genome and organ epigenome in the mouse will produce a powerful tool to unpick complex but similar mechanisms of fibrosis in man.

## Abbreviations

BSA: Bovine serum albumin; CCl4: Carbon tetrachloride; CKD: Chronic kidney disease; CTGF: Connective tissue growth factor; ECM: Extracellular matrix; EMT: Epithelial to mesenchymal transition ESRD, End stage renal disease; ESTs: Expressed sequence tags; GFR: Glomerular filtration rate; GWAS: Genome-wide association studies; HAM: Haplotype association mapping; IBD: Inflammatory bowel disease; IFN-γ: Interferon gamma; IL-1β: Interleukin 1 beta; IP: Intraperitoneal; MMP: Matrix metalloproteinase; PF: Pulmonary fibrosis; QTL: Quantitative trait loci; rhBMP: recombinant human bone morphogenetic protein; SNPs: Single nucleotide polymorphisms; TGF-β: Transforming growth factor beta; TIMP: Tissue inhibitors of matrix metalloproteinase; TNF-α: Tumor necrosis factor alpha; UUO: Unilateral ureteral obstruction.

## Competing interests

CMH is employed by Baxter Healthcare.

## Authors’ contributions

LW and SEH performed the research and the writing of the manuscript. AMS, PECB, CMH and RK edited the manuscript. PJM performed research and edited the manuscript. All authors read and approved the final manuscript.

## Authors’ information

LW is supported by BBSRC and Baxter Healthcare. SEH is supported by the University of Manchester, UK. AS and PECB are supported by the NHS, UK. CMH is employed by Baxter Healthcare. RK is employed by The Jackson Laboratory. PJM is a Canadian Institutes of Health Research clinician scientist.
